# Maternal Distress and Social Support Are Linked to Human Milk Immune Properties

**DOI:** 10.3390/nu13061857

**Published:** 2021-05-29

**Authors:** Anna Ziomkiewicz, Anna Apanasewicz, Dariusz P. Danel, Magdalena Babiszewska, Magdalena Piosek, Magdalena Orczyk-Pawiłowicz

**Affiliations:** 1 Laboratory of Anthropology, Institute of Zoology and Biomedical Research, Faculty of Biology, Jagiellonian University, Gronostajowa 9, 30-387 Krakow, Poland; anna.ziomkiewicz-wichary@uj.edu.pl; 2 Department of Anthropology, Hirszfeld Institute of Immunology and Experimental Therapy, Polish Academy of Sciences, Weigla 12, 53-114 Wroclaw, Poland; anna.apanasewicz@hirszfeld.pl (A.A.); dariusz.danel@hirszfeld.pl (D.P.D.); magdalena.babiszewska-aksamit@pan.pl (M.B.); 3 Institute of Psychology, University of Wroclaw, Dawida 1, 50-529 Wroclaw, Poland; magdalena.piosek2@uwr.edu.pl; 4 Department of Chemistry and Immunochemistry, Wroclaw Medical University, Skłodowskiej-Curie 48/50, 50-369 Wroclaw, Poland

**Keywords:** breast milk, immunoactive factors, cortisol, maternal stress, social support

## Abstract

Possible alterations of maternal immune function due to psychological stress may reflect immunoactive factor levels in breast milk. This study aimed to assess the association between maternal distress and breast milk levels of secretory IgA (SIgA), IgM, IgG, and lactoferrin (LF). We hypothesized that this association is moderated by maternal social support achieved from others during lactation. The study group included 103 lactating mothers and their healthy five-month-old infants. Maternal distress was determined based on the State Anxiety Inventory and the level of salivary cortisol. Social support was assessed using the Berlin Social Support Scales. Breast milk samples were collected to test for SIgA, IgM, IgG, and LF using the ELISA method. Milk immunoactive factors were regressed against maternal anxiety, social support, salivary cortisol, and infant gestational age using the general regression model. Maternal anxiety was negatively associated with milk levels of LF (β = −0.23, *p* = 0.028) and SIgA (β = −0.30, *p* = 0.004), while social support was positively associated with milk IgG (β = 0.25, *p* = 0.017). Neither anxiety nor social support were related to milk IgM. No association was found between the level of maternal salivary cortisol and immunoactive factors in milk. Our results suggest that maternal psychological wellbeing and social support may affect milk immune properties.

## 1. Introduction

Breastfeeding is recommended as the golden standard in newborn and infant nutrition during the first six months of life [1,2]. Besides nutritional value, breast milk is rich in immunological factors, including immunoglobulins, lactoferrin, and lysozyme, crucial for the newborn and infant immature immune system [3,4,5]. Antibodies are key factors of an adaptive immune response. Immunoglobulin G (IgG) is the primary antibody class found in the circulation [6]. However, in extracellular fluids, such as breast milk, the secretory immunoglobulin A predominates [4].

During pregnancy, of all immunoglobulins, IgG is the only antibody class that is transferred from the maternal circulation to the developing fetus across the placenta. The passive transfer of IgG is mediated by the FcRn receptor in a gestational age-related manner [7,8]. After birth, newborns are deficient in functional plasma cells capable of IgG production, but, in the first days of life, they can still use the antibodies provided during fetal life. For this reason, milk immunoglobulins, including primary secretory immunoglobulin A (SIgA), immunoglobulin M (IgM), and IgG transferred during breastfeeding, are essential for offspring during the first three months of life [9,10].

The specificity of milk immunoglobulins is determined by different pathogens with which the mother had contact before and during pregnancy [11]. In line with this, the health status of pregnant mothers shapes the immunological properties of their milk. During the first stage of lactogenesis, an increase in lactose, protein, and immunoglobulin levels is observed. Since milk production begins already at 28 weeks of normal pregnancy [12], perinatal risk factors have a substantial impact on the onset and duration of lactation, as well as on the nutritional and protective value of breast milk [13,14].

Psychological stress is a constant element of our lives that affects body function through the nervous system’s direct action and the network of secreted hormones [15]. The impact of stress, including psychological stress, on immunity is well-established [16]. The evoked immunological response can be protective, pathological, or regulatory, depending on its intensity, duration, and chronicity [17]. On the other hand, scientific data also indicate that the immune system impacts stress resistance and coping with stress [18]. Psychological stress modulates the immunological system at different levels, from regulating single immunological factors to the whole system, ultimately affecting physical health [16].

Pregnancy and the perinatal period, including successful breastfeeding, are stages of increased exposure to stress associated with the physiological course and significant energy costs of pregnancy, childbirth, and lactation. Adjustment to entirely new conditions and life roles after birth adds another psychological component of stress, the severity of which may differ depending on maternal status and social support [19,20]. Since milk serves as an important signal of the maternal physiological state passed to an infant during breastfeeding [21], possible alterations of maternal immune function in response to stress may reflect the levels of immunoactive factors in breast milk [22].

However, evidence for the effect of maternal distress on milk immune properties is mixed and limited mainly to SIgA [23,24,25,26,27,28]. Some of the previous research [23,25,27] conducted in newborns demonstrated an association between milk SIgA and different questionnaire measures of maternal mood and distress. Other studies [24,26,28] demonstrated a lack of an association between levels of milk SIgA or other immunoactive factors (IgG, IgM, interleukins, growth factors, and chemokines) and maternal distress assessed either with questionnaires or quantified by measures of cortisol.

The observed discrepancies in the study results come from several sources, including relatively small sample sizes, variable methods to assess maternal distress, and early or varying infant age. These discrepancies call for further systematic studies examining a wider range of milk immunoactive components. Furthermore, other factors with the ability to modify the maternal response to stress, like social support and coping mechanisms, should also be included. They might confound or moderate the association between maternal stress and milk immune properties. Several previous studies demonstrated that social support is related to better immune function [29], probably via buffering stress [30] and blunting stress responsiveness [31]. However, others showed that the effect of social support might be independent and not mediated via stress [32,33]. Thus, the exact physiological mechanism behind the observed associations between social support and immune function is not fully understood.

This study aims to further investigate these discrepancies by studying the association between various markers of milk immune properties (levels of SIgA, IgG, IgM, and lactoferrin) and maternal distress characterized by the state of anxiety and reactivity to a stressor measured with salivary cortisol. We studied this association in a sample of women homogenous in education, socioeconomic status, and infant age. Following previous research, we hypothesized that maternal distress would be negatively related to milk immune properties. Furthermore, the study also aims to assess the association of milk immunoactive components with maternal social support. We hypothesized that maternal social support would be positively related to milk immune properties.

## 2. Methods

### 2.1. Study Sample and Protocol

A sample of 160 mothers and their healthy infants born on-term from Wroclaw, southwestern Poland, took part in a study on the association between maternal stress and breast milk composition. Participants were recruited who met the following inclusion criteria: (1) neither mothers nor infants suffered from metabolic diseases, such as diabetes or thyroid diseases, or genetically inherited conditions; (2) infants were born from a single, uncomplicated pregnancy, with a birth weight not lower than 2500 g and not earlier than at the 37th week of pregnancy; (3) infants were fed on-demand exclusively with breast milk; and (4) at the moment of recruitment, infants were approximately five months old. In addition, for the current analysis, all mothers who suffered from any infections, including mastitis (ongoing or during the two weeks prior to the study), were also excluded due to their possible effect on the level of produced immunoactive factors [23]. Thus, the final number of participants was reduced to 103.

During the first meeting scheduled when the children were approximately five months old, the mothers signed the informed consent form, and were instructed about all study procedures. Trained study assistants performed maternal anthropometric measurements. Mothers also received a general questionnaire to be filled in at home about basic maternal demographics, such as age, birth, education, life, economic satisfaction, marital status, reproductive history, health, and basic infant characteristics, including age. They also received psychological questionnaires to assess anxiety and social support. These questionnaires were returned at the second meeting approximately one week later. At this meeting, a single milk sample was taken, and the cold pressor test [34] was conducted to assess maternal hormonal response to a mild stressor. The study protocol was approved by the Bioethical Committee of Lower Silesian Medical Chamber in Wroclaw.

### 2.2. Maternal Anxiety and Social Support

Maternal anxiety as a state and trait was assessed using the Polish version of the State-Trait Anxiety Inventory (STAI) [35]. This questionnaire is based on a four-point Likert scale, and includes 40 questions, 20 items for assessing trait anxiety and 20 for state anxiety [36].

Social support, which participants received from others, was assessed using a Polish adaptation [37] of the Berlin Social Support Scale (BSSS) [38]. The questionnaire is also based on a four-point Likert scale. The questionnaire allows for the assessment of instrumental, emotional, and informational support, as well as the perceived need for this support. In our analysis, we used the sum of the points given to different support forms with the exclusion of the need for support.

### 2.3. Milk Samples

Mothers collected milk samples in sterile containers using a Medela Symphony breast pump (Medela AG, Baar, Switzerland) at the meeting room under the supervision of the research assistant. Mothers were instructed to pump milk from one breast until empty. To standardize milk collection time against possible diurnal changes in breast milk composition, samples were collected between the second and third feeding episode of the day, where the first feeding was the one after which an infant showed daytime activity [39,40]. Since no significant difference in milk composition was found between the left and right breast [41], the mothers were free to choose from which breast the milk was collected. However, they were strongly encouraged to pump milk from the breast opposite to the one they had recently breastfed from. Immediately after collection, milk samples were stirred, portioned into smaller containers, and stored at −80 °C for later analysis.

### 2.4. Determination of Immunoglobulin and Lactoferrin Concentration in Milk

Prior to determining immunoglobulin and lactoferrin concentrations in milk, all collected milk samples were centrifuged at 3500× *g* at 4 °C for 35 min to obtain defatted milk samples. The aqueous phase of the analyzed milk samples was stored at −20 °C.

The concentrations of all analyzed immunoglobulins, namely SIgA, IgG, IgM, and lactoferrin (LF) in defatted milk (skim milk) samples, were quantified by the enzyme-linked immunosorbent assay (ELISA) using a previously developed procedure with modification [42,43,44]. In short, microtiter plates (Nunc International, Naperville, IL, USA) were used to determine all immunoglobulins and LF levels. For blocking and washing steps, TBS (pH = 7.5) containing 0.5% Tween-20 and TBS (pH = 7.5) containing 0.05% Tween-20 were used, respectively. The antibodies applied in the tests were: for IgG, F(ab’)2 fragments of goat anti-human IgG (Jackson ImmunoResearch, Europe Ltd., Ely, UK); for IgM, rabbit anti-human IgM antibody (Jackson ImmunoResearch, Europe Ltd., Ely, UK); for SIgA, mouse monoclonal anti-secretory component IgA antibodies (Sigma, St. Louis, MO, USA); and for lactoferrin, rabbit anti-human lactoferrin antibodies phosphatase-labeled (Jackson ImmunoResearch Europe Ltd., Ely, UK). Standard curves were constructed using a commercially available preparation of human serum IgG (Jackson ImmunoResearch, Europe Ltd., Ely, UK), human IgM (Jackson ImmunoResearch, Europe Ltd., Ely, UK), human colostrum IgA (Sigma, St. Louis, MO, USA), and human milk lactoferrin (Sigma Aldrich, St. Louis, MO, USA), respectively. For detection, horseradish peroxidase (HRP) and alkaline phosphatase (AP)-labeled antibodies were used, namely, for IgG, phosphatase-labeled rabbit anti-human IgG Fcγ fragment-specific antibodies (Jackson ImmunoResearch, Europe Ltd., Ely, UK); for IgM, horseradish peroxidase-conjugated goat anti-human IgM antibodies (Jackson ImmunoResearch, Europe Ltd., Ely, UK); for S-IgA, horseradish peroxidase-conjugated goat anti-mouse IgG antibodies (Sigma, St. Louis, MO, USA); and for LF, rabbit anti-human lactoferrin antibodies phosphatase-labeled (Jackson ImmunoResearch Europe Ltd., Ely, UK). The enzymatic reactions for HRP and AP were developed with appropriate substrates. The obtained absorbances were quantified at 492 nm (reference filter = 630 nm) for HRP and at 405 nm (reference filter = 630 nm) for AP using a Stat Fax 2100 Microplate Reader (Awareness Technology Inc., Palm City, FL, USA).

All defatted milk samples were quantified at three different sample dilutions dedicated to the individual parameter, each in duplicate. The calculated intra-assay and inter-assay coefficients of variation ranged from 3.2% to 4.8%; for IgM determination, from 4.1 to 3.6%; for SIgA determination, from 2.8 to 3.3%; and for LF determination, from 4.3% to 3.6%, respectively.

### 2.5. Salivary Samples

The hand cold pressor test (CPT) [34] was performed to assess maternal physiological reactivity to a mild stressor. Women were asked to immerse the hand in ice water for one minute. Four saliva samples to measure cortisol level (Csal) were taken (1) 10 min before, (2) 1 min before, (3) immediately after, and (4) 10 min after the test. The average level of Csal measured in all saliva samples was calculated to include in further analysis.

### 2.6. Cortisol in Saliva

Samples were collected in sterile 1 mL Eppendorf tubes and stored at −80 °C until the assay. After thawing and centrifugation (1500× *g* for 10 min), samples were tested for salivary C concentration using enzyme-linked immunosorbent assays (Salivary Cortisol ELISA, DRG Instruments GmbH, Marburg, Germany) according to the manufacturer’s recommendations. The samples were analyzed in duplicate, and the average intra-assay coefficient of variation for cortisol was less than 4.5%.

### 2.7. Statistical Analysis

The distribution of milk immunoglobulins (SIgA, IgM, and IgG) and salivary cortisol (Csal) were diverted from normal; thus, all of their values were log-transformed to assure normality. Possible differences between women delivering vaginally and via cesarean section (C-section) in levels of immunoactive factors and maternal and infant characteristics were tested with Student’s t-test. Linear associations between immunoactive milk components and potential confounding variables (maternal age, BMI, child age, gestational age, economic, and life satisfaction) were tested using Pearson correlations.

The association between maternal anxiety, social support, and milk immunological properties was analyzed using the general regression model. Each immunoactive factor (LF, SIgA, IgM, and IgG) was regressed against maternal anxiety, logCsal, and social support, together with infant age, gestational age, and maternal BMI included as additional predictors in multiple regression separate models. For each model, collinearity was tested using the Durbin−Watson test for autocorrelation. All statistical analyses were performed using Jamovi version 1.6 (2020) [45].

## 3. Results

Out of 103 women (mean age = 31.1 ± 4.09) included in the analyses, almost 60% were primiparous; thus, the mean parity was 1.5 ± 0.65. Most women (over 80%) had a higher education, confirmed by at least a bachelor’s degree, and relatively high life and economic satisfaction (median = 6.0 out of 7.0 possible points). All women lived with their partners or husbands, were non-smokers, and did not drink alcohol.

No statistical differences in maternal and infant characteristics were found between women delivering vaginally and via C-section, except for the level of anxiety. Women who delivered vaginally had a higher level of anxiety than women who delivered via C-section (t_(101)_ = −2.19, *p* = 0.031). General characteristics of the total sample and the group of women delivering vaginally vs. via C-section are listed in Table 1.

The exploratory analysis for potential confounding effects demonstrated no significant correlation between maternal age and BMI, life and economic satisfaction, or levels of milk immunoactive factors (Table S1). In contrast, a significant correlation was found for infant gestational age. Gestational age correlated negatively with lactoferrin (*r* = −0.30, *p* = 0.002) and SIgA (*r* = −0.20, *p* = 0.045). Based on these results, gestational age was included as an additional covariate in all further analyses.

The maternal level of anxiety correlated negatively with the level of social support (*r* = −0.38, *p* < 0.001), while neither anxiety (*r* = −0.11, *p* = 0.283) nor support (*r* = 0.03, *p* = 0.764) correlated with the level of Csal.

### Relationship between Maternal Distress, Social Support, and Milk Immunoactive Factors

**Figure 1:**
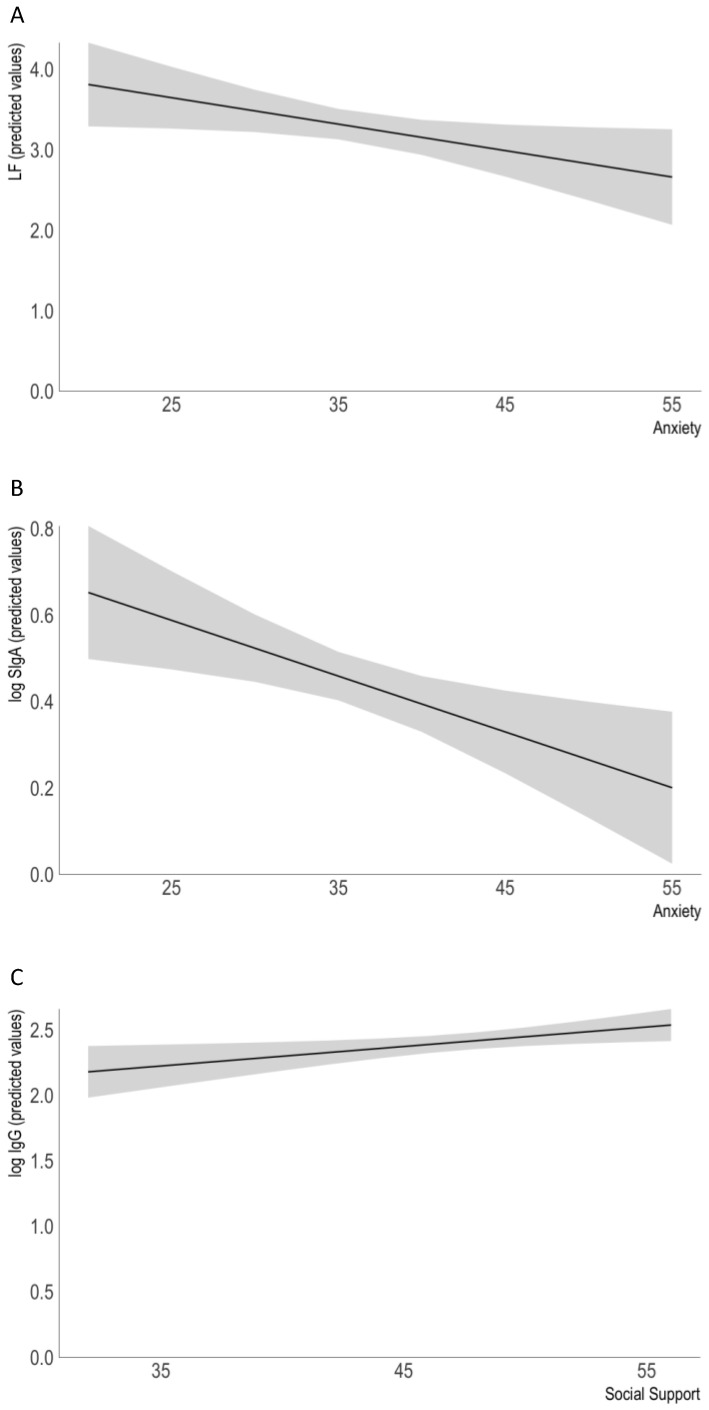
The association between maternal state of anxiety and social support, and the predicted level of immunoactive components in human milk (estimated marginal means, adjusted for the means of other variables in the models). A negative association was found between the level of lactoferrin (**A**) and SIgA (**B**) and maternal anxiety. A positive association was found for the level of IgG and social support (**C**).

Maternal distress and social support were significantly associated with milk immunoactive factors (Table 2, Figure 1). In particular, maternal anxiety (β = −0.23, *p* = 0.028) was negatively related to the level of lactoferrin, but no association was found with social support (β = −0.03, *p* = 0.741). Another significant covariate negatively predicting the level of lactoferrin was infant gestational age (β = −0.30, *p* = 0.002). The whole model, which included postnatal and gestational age, maternal BMI, and salivary cortisol levels, explained around 12% of the variance in lactoferrin levels.

Maternal anxiety (β = −0.30, *p* = 0.004) was also negatively related to the level of SIgA in milk, and the association with social support was only marginally significant (β = −0.19, *p* = 0.06). Another factor that predicted the level of SIgA was infant gestational age (β = −0.19, *p* = 0.043). All factors included in the model (gestational and postnatal age, maternal BMI, and salivary cortisol level) together explained 11% of the variance in the level of SIgA.

In contrast, only social support (β = 0.25, *p* = 0.017) was related to the level of IgG in milk, while no association was found for maternal anxiety (β < 0.01, *p* = 0.969). The level of IgG was also positively related to postnatal infant age (β = 0.28, *p* = 0.006). All factors included in the model (gestational and postnatal age, maternal BMI, and salivary cortisol level) explained around 9% of the variance in IgG levels.

Neither maternal anxiety (β = −0.19, *p* = 0.079) nor social support (β = 0.12, *p* = 0.259) predicted IgM level in milk. The whole model, which included gestational and postnatal age, maternal BMI, and salivary cortisol level, was statistically not significant. Table 2 presents the detailed results of all regression models.

## 4. Discussion

This study describes the association between maternal distress, social support, and breast milk immunoactive properties. In particular, it demonstrates a positive relationship between the mother’s social support and the level of IgG in her milk. Furthermore, it supports the previously described negative association between maternal anxiety and levels of milk SIgA, but also shows that anxiety may be linked to the level of other immunoactive factors, such as milk lactoferrin.

To our knowledge, this is the first study demonstrating that maternal social support is associated with immunoactive compounds of breast milk. Previous research showed that higher support from others was associated with earlier breastfeeding initiation and a longer period of total breastfeeding [46,47,48,49,50]. Our preliminary results also show that the number of people who helped lactating mothers was positively related to the content of polyunsaturated fatty acids (PUFAs) in milk [51]. Physiological mechanisms beyond this effect might include the reduction of chronic psychosocial stress associated with motherhood. The role of long-term and chronic psychosocial stress in impairing immune function has been widely recognized [52]. Several studies indicated decreased blood or saliva immunoactive factors in response to chronic stress [53].

On the other hand, studies demonstrated that social support modifies the stress response. In particular, Heinrichs and others (2003) [31] reported that social support decreased cortisol levels in response to the TSST (Trier Social Stress Test), an experimental procedure to induce high psychosocial stress. Higher social support was also positively related to the level of antibodies, including IgG and IgM, after different vaccinations [54]. These results are of particular significance to our study, which showed a positive association between maternal social support and milk IgG levels. Apart from the local synthesis in the mammary gland, the milk level of IgG is supported by the active transfer of IgG from maternal serum [55]. Thus, the higher level of IgG in milk is probably an indicator of a higher IgG level in maternal serum. This result suggests that women with higher social support could produce a higher level of immunoglobulins and simultaneously experienced lower anxiety in response to psychological challenges. While social support and anxiety were significantly and negatively correlated, a positive association between social support and milk IgG may reflect the common pathway of interaction between stress, social support, and immune function. However, the exact physiological mechanism linking social support and milk immunoactive properties requires further identification.

Our study found a negative association between the state of anxiety and levels of SIgA, which is the main milk immunoglobulin. This result corroborates some of the previous works demonstrating the negative effect of maternal distress on milk immunoactive properties, and remains in agreement with general theoretical predictions and experimental observations that the chronic experience of psychological symptoms, such as anxiety, may downregulate the immune system [56].

Accordingly, Kawano and Emori (2015) [25] found a similar negative association between maternal anxiety state and milk SIgA in Japanese mothers on the second or third day postpartum. Our results suggest that this effect may extend to the later stages of breastfeeding. Moreover, the level of maternal anxiety observed in this population was similar to ours (mean score = 39.6 vs. 36.1).

Interestingly, a large recent study by Aparicio and others (2020) [28] found no effect of maternal psychological distress (including anxiety) on the level of milk SIgA or any other analyzed immunoactive factors. Compared to our study, this lack of association might result from differences in the milk collecting protocol (foremilk samples vs. whole milk samples), lower infant age (two, six, or 12 weeks vs. five months), different geographical location [57], and decreased levels of maternal anxiety (median score = 27.0 vs. 36.3). The differences in the observed level of anxiety between the studies may suggest that the negative relationship with SIgA is revealed only after a specific maternal anxiety threshold is reached.

It has to be acknowledged that anxiety (either state or trait) describes an individual’s subjective feelings when experiencing stressful events. These subjective feelings may not necessarily represent physiological responses to stressors and, in turn, possible changes in breast milk composition. Thus, analyzing the association between anxiety or perceived stress and milk composition requires controlling for objective physiological markers of stress reactivity or stress response (e.g., cortisol level). On the other hand, studies of women during pregnancy and the postpartum period demonstrated significant positive associations between psychological distress operationalized by different measures (anxiety, life events, and perceived stress). Participants who experienced more types of prenatal psychological distress were at higher risk for developing more severe physiological outcomes, such as depression, during the postnatal period [58].

The specificity of transferred milk immunoglobulins is limited to the range of pathogens with which the mother’s immune system had contact previously [11,59]. Milk immunoglobulin composition, especially in a qualitative context, i.e., specificity, might be shaped before and during pregnancy by the previous infection, vaccination, and the mother’s allergies [60]. In turn, the overall effect of psychosocial factors on the level of milk immunoglobulins is modest. However, stress-related, low-grade inflammation may interfere with the normal function of immune B cells [61] and modulate the production of all immunoglobulins, or only their particular class.

The results of our analysis also demonstrated a significant negative association between maternal distress and the level of LF. Little is known about the external factors that influence the level of LF in human milk. Previous studies identified lactation stage [62], maternal age [63], and ethnicity [64] as the main factors regulating variation in the level of milk LF. Our research identifies maternal anxiety as yet another factor. Although no such analysis has been published before, experimental studies in rodents and humans suggest a possible physiological base for this association. Single-dose oral administration of bovine LF was found to mitigate the physiological response to experimentally induced psychological stress in rats [65] and humans [66]. Thus, it is possible that in breastfeeding mothers with increased anxiety, LF is preferentially used to alleviate the consequences of higher stress, and LF transfer to milk remains low.

The principal strength of this study lies in a well-characterized and homogenous cohort of mothers with stabilized fifth-month lactation. A very homogenous cohort in terms of residence eliminated the possible impact of geographical location on the immunological quality of the analyzed milk [57,67]. Furthermore, all mothers in the cohort were non-smokers and had a relatively high socioeconomic status indicated by attained education and economic satisfaction. Both smoking and economic status were previously found to affect immune function and specifically immunoglobulin levels in human serum and saliva [68,69]. These factors might affect the levels of immunoglobulins in breast milk and confound the association between maternal distress and milk immunactive properties. However, a single sample collection protocol and observational character may limit the study’s generalizability to a single time point during lactation. Future studies should also seek to include mothers from the general population with a broader socioeconomic status and education range.

## 5. Conclusions

In summary, the evidence from our study suggests that the mother’s psychological wellbeing may be associated with the immunological properties of her milk. Although evidence from the literature is mixed, the study suggests that increased anxiety, most probably associated with higher psychological stress, negatively affects milk immune properties. In contrast, social support, which was demonstrated to mitigate the stress response and strengthen the individual immunological response to challenges, may also boost milk properties. Noteworthy is the fact that both factors interacted with different markers of milk immune properties, which may suggest different physiological pathways of the observer associations. Since maternal wellbeing is crucial for ensuring the adequate immune protection of their breastfed children, the results of our study advocate intensifying social support of mothers during pregnancy and breastfeeding as an overarching public health goal.

In contrast, only social support (β = 0.25, *p* = 0.017) was related to the level of IgG in milk, while no association was found for maternal anxiety (β < 0.01, *p* = 0.969). The level of IgG was also positively related to postnatal infant age (β = 0.28, *p* = 0.006). All factors included in the model (gestational and postnatal age, maternal BMI, and salivary cortisol level) explained around 9% of the variance in IgG levels.

Neither maternal anxiety (β = −0.19, *p* = 0.079) nor social support (β = 0.12, *p* = 0.259) predicted IgM level in milk. The whole model, which included gestational and postnatal age, maternal BMI, and salivary cortisol level, was statistically not significant. Table 2 presents the detailed results of all regression models.

## Figures and Tables

**Table 1:** General characteristics of all study participants and differences in general characteristics between women delivering vaginally and via C-section.

	All (*n* = 103) $\bar{x}\left(SD\right)$	C-Section (*n* = 40) $\bar{x}\left(SD\right)$	Vaginal (*n* = 63) $\bar{x}\left(SD\right)$
Mothers			
Age	31.1 (4.09)	30.3 (3.46)	31.3 (4.32)
Education (% higher)	84.5%	87.5%	81.0%
BMI (kg/m^2^)	23.0 (3.45)	22.9 (2.86)	22.9 (3.37)
Anxiety	36.1 (6.85)	34.2 (7.89) *	37.4 (5.85) *
Social support	47.3 (5.86)	47.9 (6.37)	47.2 (5.33)
Csal (ug/dL)	1.93 (1.57)	1.74 (1.150)	2.25 (1.930)
Infants (57 boys, 46 girls)			
Age (months)	4.8 (0.54)	4.9 (0.42)	4.7 (0.59)
Gestational age (weeks)	39.8 (1.38)	39.8 (1.39)	39.8 (1.30)
Birth weight (kg)	3.53 (0.434)	3.55 (0.47)	3.51 (0.38)
Birth length (cm)	54.7 (2.74)	55.2 (2.88)	54.4 (2.55)
Breast milk			
Lactoferrin (g/L)	3.28 (1.020)	3.20 (1.087)	3.33 (0.837)
SIgA (g/L)	1.62 (0.458)	1.55 (0.427)	1.65 (0.837)
IgG (mg/L)	11.70 (4.040)	11.47 (3.812)	11.95 (4.022)
IgM (mg/L)	2.38 (0.210)	2.40 (1.586)	2.17 (1.756)

* *p* < 0.05. BMI—Body Mass Index, Csal—salivary cortisol

**Table 2:** Results of the regression models for the association between maternal distress (anxiety, logCsal), social support, and levels of milk LF, SIgA, IgG, and IgM.

	Standardized Coefficient β	95% CI for *β*	*p*
LF (F_(6,96)_ = 3.27, *p* = 0.006, R^2^_adj_ = 0.12)			
Anxiety	−0.23	−0.433 to −0.025	0.028
Social support	−0.03	−0.237 to 0.169	0.741
logCsal	−0.09	−0.279 to 0.103	0.364
Infant age	0.14	−0.058 to 0.334	0.167
Gestational age	−0.30	−0.485 to −0.110	0.002
Maternal BMI	0.12	−0.068 to 0.311	0.207
log SIgA (F_(6,96)_ = 3.18, *p* = 0.007, R^2^_adj_ = 0.11)			
Anxiety	−0.30	−0.509 to −0.010	0.004
Social support	−0.19	−0.399 to 0.008	0.060
logCsal	0.12	−0.068 to 0.315	0.204
Infant age	0.14	−0.060 to 0.334	0.170
Gestational age	−0.19	−0.381 to −0.006	0.043
Maternal BMI	0.15	−0.041 to 0.339	0.123
log IgG (F_(6,96)_ = 2.61, *p* = 0.022, R^2^_adj_ = 0.09)			
Anxiety	0.01	−0.203 to 0.212	0.969
Social support	0.25	0.045 to 0.458	0.017
logCsal	0.15	−0.0436 to 0.345	0.127
Infant age	0.28	0.081 to 0.480	0.006
Gestational age	0.12	−0.072 to 0.308	0.222
Maternal BMI	0.11	−0.083 to 0.303	0.260
log IgM (F_(6,96)_ = 1.74, *p* = 0.120, R^2^_adj_ = 0.04)			
Anxiety	−0.19	−0.402 to 0.023	0.079
Social support	0.12	−0.091 to 0.333	0.259
logCsal	0.05	−0.149 to 0.249	0.620
Infant age	0.09	−0.113 to 0.296	0.375
Gestational age	0.06	−0.137 to 0.253	0.556
Maternal BMI	0.17	−0.032 to 0.3630	0.099

## Data Availability

The data presented in this study are available on request from the corresponding author. The data are not publicly available due to ongoing analysis.

## Supplementary Materials

The following are available online at https://www.mdpi.com/article/10.3390/nu13061857/s1, Table S1: Correlation coefficients for the association between milk immunoactive factors (LF, SIgA, IgG, IgM) and potential confounding factors.
