# Supplementary material for: Maternal Distress and Social Support Are Linked to Human Milk Immune Properties
Source: Nutrients. 2021 May 29;13(6):1857. doi: 10.3390/nu13061857 (PMC8226629; doi:10.3390/nu13061857)
Supplement: Supplementary file 1 [file nutrients-13-01857-s001.zip › nutrients-1202898-SI.pdf]

Supplementary Materials

Table S1: Correlation coefficients for the association between milk immunoactive factors (LF, SIgA, IgG, IgM) and potential confounding factors

	LF		lnSIgA		lnIgG		lnIgM	
	r	p	r	p	r	p	r	p
Maternal age	0.02	0.804	0.09	0.382	-0.03	0.751	-0.18	0.070
Maternal BMI	0.06	0.560	0.09	0.355	0.06	0.544	0.13	0.183
Child age	0.17	0.08	0.11	0.275	0.17	0.075	0.03	0.764
Gestational age	0.30	0.002	0.20	0.045	0.09	0.372	0.06	0.511
Economic Satisfaction	0.03	0.794	0.19	0.055	0.04	0.700	0.18	0.067
Life Satisfaction	-0.15	0.122	0.04	0.680	0.04	0.710	0.19	0.059
